# The Wallpaper Effect: The Contact Hypothesis Fails for Minority Group Members Who Live in Areas with a High Proportion of Majority Group Members

**DOI:** 10.1371/journal.pone.0082228

**Published:** 2013-12-11

**Authors:** Fiona Kate Barlow, Matthew J. Hornsey, Michael Thai, Nikhil K. Sengupta, Chris G. Sibley

**Affiliations:** 1 School of Psychology, The University of Queensland, Brisbane, Queensland, Australia; 2 Department of Psychology, The University of Auckland, Auckland, New Zealand; University G. d'Annunzio, Italy

## Abstract

We aim to provide one explanation for why the link between contact and prejudice is consistently less strong for minority group members than it is for majority group members. Specifically, we propose a “wallpaper effect” such that contact works to increase minority group members' positivity towards majority groups when they live in areas densely populated with other minority group members. Conversely, we suggest that when minority group members live in neighborhoods patterned with majority group faces (as is so often the case), contact will be less transformative. We test this assumption using a large sample of both New Zealander minority (Māori; *N* = 925) and majority (European; *N* = 3805) group members. In line with predictions, Māori who lived in minority dense neighborhoods showed the traditional association between contact and increased warmth towards New Zealander Europeans. This relationship, however, was weak or non-existent when they lived in primarily European neighborhoods. Contact effects in majority group members were unaffected by neighborhood composition. The interaction held when controlling for, and was not explained by: gender, income, experiences of harm, cognitions of race-based rejection, or realistic threat. We provide the first evidence to suggest that when it comes to minority group members' intergroup attitudes, contact with majority group members may be a relatively ineffective predictor *unless* the “wallpaper” of their lives is minority-dense.

## Introduction

Positive contact with members of outgroups typically leads to improved intergroup attitudes [1], [2]. There are limits, however. In particular, the power of contact to improve intergroup attitudes is predominantly evident for members of the comparatively privileged *majority* group. For traditionally disadvantaged *minority* group members, the association between contact and outgroup attitudes is less clear and consistently less strong [2]. We seek to better understand the differential impact of intergroup contact on majority and minority group members by proposing neighborhood composition (i.e., the proportion of the neighborhood made up of ethnic minorities) as a moderator of the relationship between contact and attitudes. Specifically, we propose a “wallpaper effect” such that for minorities, contact becomes less predictive of intergroup attitudes the higher the ratio of outgroup members in one's neighborhood. To the extent that there is a relative scarcity of outgroup members (as is typically the case for majority group members) contact will predict intergroup attitudes. But to the extent that one is surrounded by outgroup members (as minority group members typically are) levels of contact become a weak or non-significant predictor of intergroup attitudes. We test our predictions in a large, representative sample of European (majority group) and Māori (minority group) New Zealanders.

### The contact hypothesis

The contact hypothesis, popularized by Gordon Allport [3], is arguably the most influential theory of prejudice reduction. According to this hypothesis, contact between groups improves intergroup attitudes, providing that the contact is characterized by equal status between group members, common goals, cooperation, and the support of relevant authorities. This basic prediction has been confirmed across a prodigious array of correlational and experimental studies [1].

In recent years researchers have been moving away from the traditional contact hypothesis to investigate a special type of contact – cross-group friendship [4]. Cross-group friendship organically meets three of the four conditions set out by Allport [3], although note that cross-group friendships will often not have the support of relevant authorities. People in a cross-group friendship can gain intimate knowledge of one another both as individuals *and* group members [5], as their friendship develops through extended contact marked by openness, positivity, and reciprocal self-disclosure [6], [7]. Meta-analyses demonstrate that much of the improvement in intergroup attitudes associated with contact is driven by the formation of cross-group friendships, and that the positive effects of contact are stronger when that contact is in the context of a friendship [1].

If intergroup contact (and in particular cross-group friendship) is effective at reducing prejudice, then it makes sense that the bulk of contact work has focussed on looking at one particular side of the contact relationship. That is, at how intergroup contact is experienced by, and influences, members of a group that have typically displayed prejudice. In the context of Black and White relations in the Western world, for example, Black people have typically been the target of White racism. Thus, we look at intergroup contact as a means by which to reduce prejudice in majority groups (in this example, White people). This is a worthwhile area of study, and critically important, but it means that the perspective of disadvantaged minority groups in contact situations has tradtionally been overlooked. While there is markedly less research looking at cross-group contact and friendship in minority groups, existing research suggests that intergroup contact is experienced differently by majority and minority group members.

### Contact in minority and majority groups

When contrasting the effects of contact in majority and minority groups, perhaps the most consistent pattern that emerges is a difference in the magnitude of association. In a meta-analytic test of the contact hypothesis, Tropp and Pettigrew [2] found that minority groups showed a weaker average relationship between outgroup contact and prejudice (*r = *−.18) than did majority groups (*r = *−.23). Tropp and Pettigrew [2] noted that this could *not* be explained by the quality of the intergroup contact – even optimal contact showed weaker associations with prejudice in minority compared to majority groups.

Two main explanations for this minority-majority asymmetry have been proposed. Tropp and Pettigrew [2] suggested that conflicting socio-historical contexts may account for the observed difference. Majority group members, who rarely consider their group status or identify themselves as ‘majority’, are mainly concerned about appearing prejudiced in intergroup settings. Thus, positive contact works effectively by alleviating these concerns. For minority group members, conversely, their main concern is being the target of prejudice [8], [9]. Here, single incidences of positive contact may be colored by co-occurring incidences of prejudice and discrimination. An alternative (but related) explanation is that, on average, minority group members are simply treated more poorly in contact situations than are majority group members. In line with this assertion, Tropp [10] demonstrated that Black Americans who perceived little discrimination showed a strength of association between contact and perceptions of interracial closeness with White Americans similar to that usually found in majority groups. By and large, however, factors that explain minority-majority asymmetry in contact effects remain untested, and rely more on intuition than empiricism.

### Prejudice and neighborhood ethnic composition

In this paper we propose and test a novel explanation for the minority-majority asymmetry – the ethnic and racial makeup of people's immediate environment. Intergroup contact was originally theorized in the context of segregation, where inter-racial socializing was relatively rare. When people who have not previously interacted with one another come together, their preconceived notions are challenged [11]. For example, people who have relatively little contact with outgroup members typically report a battery of fears and anxieties centering around how the outgroup will respond to them. Contact can allay such fears, and consequently reduce anxiety and prejudice [11]. This is especially the case if the outgroup member that they interact with is seen as typical of, and representative of, the outgroup [5], [12].

What might happen, though, in contexts where there is already a degree of racial diversity in a neighborhood? Extending from meta-analyses showing that quantity of intergroup contact reduces prejudice [1], we might expect to see neighborhoods with relatively diverse populations showing lower levels of prejudice than ethnically homogenous neighborhoods. This is not always the case, however. Much work has been done on the link between the number of outgroup neighbors, sometimes referred to as *opportunity for contact* [13], and attitudes towards different outgroups. For majority group members, the issue of neighborhood racial diversity is a vexed one. Past research generally suggests that as minority group proportion increases, so too does prejudice [14]–[16] – a finding usually attributed to increased group-based threat posed by an increasing minority [17], [18]. For example, Ayers, Hofstetter, Schnakenberg and Kolody [19] found that White Americans living in greater proximity to Latino populations also reported increased opposition to legal Latino immigration and amnesty for illegal immigrants who had lived in the country for four or more years. Likewise, in his work looking at conflict between Romanians and Hungarians in Transylvania, Cernat [14] argued that under adverse conditions, the presence of outgroup members in one's immediate neighborhood could be a recipe for conflict rather than cooperation. In terms of contact research, a number of studies have found opposing effects for opportunity for contact and actual contact. Specifically, number of outgroup neighbors has been shown to predict increased threat perceptions and consequently prejudice, while at the same time cross-group friendship predicts reduced prejudice [20].

While the majority of available research suggests that regional diversity or minority group proportion is linked to increased prejudice, it should be noted that this pattern is not universal. Sometimes traditional contact, rather than threat effects are found. For example, some census level data in the United States indicates that as the percentage of the neighborhood that is made up of Black people increases, so too do minority group perceptions of racial discrimination decrease [21]. This finding supports an ethnic density hypothesis, whereby mostly Black contexts breed the lowest levels of discrimination. Research from Italy also shows that Italian people living in neighborhoods with a higher proportion of Black immigrants also report more favorable attitudes and emotions about Black immigrants [22]. Similarly, Wagner, van Dick, Pettigrew and Christ [23] also found that people living in East Germany had fewer opportunities for contact with members of ethnic minority groups, and as a consequence, reported higher levels of prejudice. Despite these findings the bulk of the research suggests that increased diversity is linked to an increase in prejudice in majority groups [14]–[16]. As such, in the present study we hypothesise that majority group members living in neighbourhoods with a high proportion of minority group members should display less warmth towards them.

In terms of the relationship between regional diversity and intergroup attitudes for members of minority groups, little is known. What we do know, however, is that from grade school onwards, minority group members report more intergroup contact than majority group members [24]. This makes sense. As a mere function of the fact that majority groups are usually just that - a numerical majority - they will experience less contact with minority group members than minority group members will experience with them. For both majority and minority group members in Western nations, then, the “wallpaper” of their social experience is White (that is, characterized by majority group faces). Irrespective of where they physically reside, majority group members inhabit a psychological space in which the (minority) outgroup is visually and culturally marginalized. Contact with minority group members, therefore, has the potential to dramatically influence their perceptions of intergroup relations, and minority outgroups in general.

For minority group members, however, contact with majorities may be routine or commonplace. In the case where minority group members are bombarded with intergroup contact (maybe the majority of their work colleagues, acquaintances, and friends are majority group members), this contact will cease to be remarkable, and hence becomes less transformative. Thus, we propose that the ratio of majority to minority group members in minority group members' neighborhoods should moderate the contact-prejudice relationship. In situations where minority group members live in neighborhoods primarily comprising other minority group members, we would expect the contact-prejudice relationship to be relatively strong (as it typically is for majority group members). But when minority group members live in neighborhoods densely populated with majority group members, we would expect the contact-prejudice relationship to be weak or non-existent.

For majority group members we would not expect the same pattern. Majority group members, irrespective of where they live, typically exist in a society in which the media, arts, entertainment, places of work and places of education are populated with majority group faces. As such, it would rarely be the case that majority members would experience the kind of “wallpaper effect” that minority group members might experience. Thus, one would almost always expect intergroup contact to retain its transformative power, implying a direct main effect of contact on intergroup attitudes rather than a moderated effect.

We propose a pragmatic, numerical explanation for our moderated finding: Contact will only be remarkable for minority group members if they live in neighborhoods with a high proportion of fellow minority group members. It is also possible, however, that minority group members who live surrounded by majority group members experience more racism, and that this negative experience trumps the effects of cross-group friendship in defining their attitudes toward the majority group. If this is the case, indices of expectations and experiences of racism should fully mediate the relationship between the outgroup proportion-contact interaction and outgroup attitudes for minority group members. Likewise, past research has found that threat is associated with outgroup proportion [17], and as such, minority group members in majority dense neighborhoods might experience more threat, and consequently threat might explain a moderated relationship. Conversely, if our original hypothesis is correct, the link between the interaction and warmth would hold even when controlling for such measures.

## The Present Study

In our study we used a large representative sample of European and Māori New Zealanders. In both groups, we measured hours spent with outgroup friends per week (cross-group friendship), and warmth towards the outgroup as the dependent variable. We also coded participants' responses by neighborhood, allowing us to assess objective, census level data of the percentage of the neighborhood population that was made up of fellow minority group members (or in the case of European New Zealanders, fellow majority group members).

To ensure that the hypothesized relationships were not just products of extraneous variables, we controlled for income, education, gender, experiences of active harm (e.g., how often participants been physically threatened), cognitions of race-based rejection (that is, expectations of being rejected on the basis of your race), and perceptions of realistic, resource-based threat [11], [25]–[27]. Experiences of active harm and cognitions of race-based rejection were both used as proxy measures of perceived discrimination.

## Methods

### Ethics statement

The study was approved by The University of Auckland Human Participants Ethics Committee. Prior to participating in the study participants were given an information sheet detailing the purpose of the study, what was involved in participation, how long data would be stored for, and how data would be used. Participants then provided signed consent. No data was retained or analyzed without signed consent.

### Participants

This study analyzed data from the 2009 New Zealand Attitudes and Values Survey (NZAVS-2009). The NZAVS-2009 is nationally representative, and sampled a total of 6,518 participants. We restricted our analyses to 4730 (72.6%) of the total sample who identified as New Zealander European (N = 3805) or Māori (N = 925) and who also listed their full residential address, and thus from whom we were able to match individual responses to minority group proportion information. This subsample contained 1911 men and 2819 women with a mean age of 47.85 (SD = 15.50).

### Questionnaire measures

***Cross-group contact*** with Māori and New Zealander Europeans was assessed by a single open-ended question asking participants how many hours they spent with friends from the other ethnic groups in the last week (minimum = 0, maximum = 168). In order to make coefficients more readily interpretable we coded these data to create an index of contact in 10-hour units that participants spent with friends from the other ethnic group. Meta-analytic findings suggest that operationalizing contact in terms of *time spent* with outgroup friends yields stronger effects than other measures, such as “felt closeness” or the number/percentage of outgroup friends [4]. Further, measures of cross-group friendship routinely include estimates of time spent with outgroup friends [11], and this particular measure has been successfully used in other studies [28], [29].

***Minority group proportion*** was recorded using census level data. Census area unit information was available for 1760 areas of New Zealand with a usually resident population. Our sample of participants spanned 1370 (77.8%) of these area units, with an average of 3.49 people per unit (*SD* = 2.45, *median* = 3). According to 2006 census data, the average number of usual residents per unit for our sampled units was 2684 people (SD = 1491), which ranged in population size from 87 to 9027 people. The proportion of the population that were Māori ranged from 1.18% to 94.76% (*M* = 15.20, *SD* = 13.78). It is this proportion that was used as a measure neighborhood composition for minority group members. Conversely, the analogous measure used for majority group members was proportion of New Zealander European. The proportion of the population that were New Zealander European ranged from 8.60% to 89.57% (*M* = 70.17, *SD* = 14.81).

***Warmth*** toward Māori and New Zealander Europeans was assessed using Affect Thermometer Ratings modeled on the US National Election Study surveys. Participants rated their feelings of warmth toward the social groups “Māori” and “New Zealand Europeans” on a scale from 1 (least warm) to 7 (most warm). We coded these data to create an index of outgroup warmth (participants' ratings of warmth toward members of the other ethnic group).

***Income*** was measured by a single open-ended item “Please estimate your own personal earnings (before tax) for the year 2009”. As data were not normally distributed, a logarithmic transformation was performed. Missing values were replaced with the logarithmic series mean.

***Experiences of active harm*** were measured via a 3-item original scale. It began with a question that asked “In your day-to-day life, how often do people in New Zealand act towards you in the following ways?” (1 = have never experienced this, 4 = sometimes experience this; 7 = often experience this). The items were: “… do things to threaten you”, “… make threatening gestures toward you” and “… attack you, or make you fear that they might.” (α = .81).

***Cognitions of rejection*** were measured via a single item adapted from Barlow and colleagues [11]: “People from other races would be likely to reject me on the basis of my race.” (1 = strongly disagree, 7 = strongly agree).

***Realistic threat*** was measured via a single item adapted from Bobo [27], which tapped into perceptions about the extent to which each group thought that the other presented a realistic threat to resources. New Zealander Europeans responded to the item: “In my opinion, more good jobs for Māori means fewer good jobs for members of other groups in New Zealand” (1 = strongly disagree, 7 = strongly agree). Māori responded to the same item, but with New Zealander Europeans as the target outgroup.

## Results

**Figure 1:**
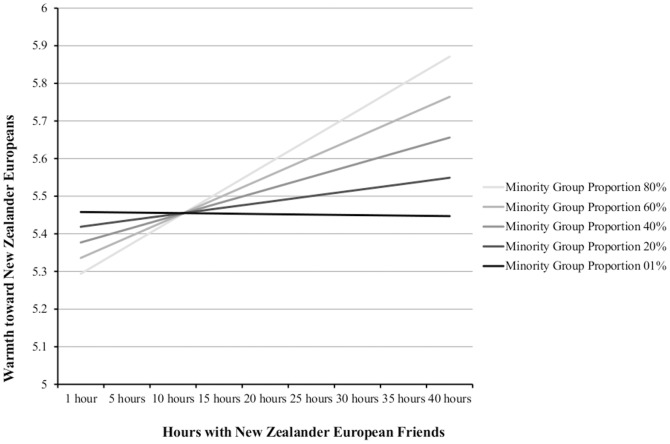
Simple slopes for Māori for the relationship between hours spent with outgroup (New Zealander European) friends on outgroup warmth depending on minority group proportion of immediate neighborhood.

Means, standard deviations, and intercorrelations between variables can be found in Table 1 for both Māori and New Zealander Europeans. We conducted a series of Multi-level Random Coefficient Models separately for our samples of Māori (925 participants nested within 623 area units, with an average of 1.485 people per unit) and European New Zealanders (3805 participants nested within 1267 area units, with an average of 3.003 people per unit). We examined whether the proportion of ingroup members living in regions interacted with hours of cross-group friendship to predict outgroup warmth. We also included the (grand mean centered) within-group effects of gender, age, income, cognitions of rejection from other ethnic groups, experiences of active harm, and perceived realistic threat. We modeled all within-group slopes as random effects and used Maximum Likelihood with robust standard error estimation. Results held when including only the key effects of interest: hours of contact with outgroup friends, the regional proportion of ingroup members, and the cross-level interactions of regional proportion and outgroup contact on intergroup attitudes. In Tables 2 and 3 results from models both including and excluding control variables can be found. Results presented below, and slopes in Figure 1, are from the full model with all control variables included.

**Table 1:** Bivariate Correlations (Māori on Lower Diagonal, New Zealander Europeans on Upper Diagonal), Means and Standard Deviations for both Māori and New Zealander Europeans.

	1.	2.	4.	5.	6.	7.	8.	9.
1. Contact with Outgroup	-	.12***	.10***	.02	−.02	.10***	.03	−.01
2. Māori (lower)/European(upper) Proportion	−.01	-	.01	.01	.05**	−.06***	−.03	−.02
4. Warmth toward Outgroup	.08*	.02	-	−.07***	.00	−.13***	−.15***	−.27***
5. Gender (0 = Female, 1 = Male)	.08*	−.06	−.04	-	.06***	.14****	.11***	.03*
6. Income (logarithmic)	−.02	−.22***	−.05	.03	−	−.08***	−.06**	−.10***
7. Active Harm	.02	.09**	−.04	.06	−.09**	-	.22***	.14***
8. Cognitions of Race-Based Rejection	.04	.12***	−.10**	.10**	−.12***	.22**	-	.20***
9. Realistic Threat Perceptions	.02	.10**	−.18***	.05	−.08*	.13**	.26***	-
***Note*** **.** *p<.05, **p<.01, ***p<.001								
**Māori**								
*Mean*	25.68	.21	5.52	.38	10.95	2.37	3.10	3.24
*Standard Deviation*	33.56	.17	1.23	.49	.71	1.15	1.82	1.77
*Range*	0–168	.02–.95	1–7	0–1	5.52–13.22	1–7	1–7	1–7
**New Zealander European**								
*Mean*	4.85	.72	4.74	.41	11.13	2.05	2.58	2.62
*Standard Deviation*	14.99	.12	1.38	.49	.69	.98	1.54	1.63
*Range*	0–168	.09–.90	1–7	0–1	6.91–14.15	1–7	1–7	1–7

**Table 2:** Model Statistics for Māori, Predicting Warmth Towards New Zealander Europeans.

	*b*	*se*	*t*
***Step 1***			
Intercept	5.518	.041	134.050***
Contact with Europeans	.029	.012	2.357**
Proportion of Māori in Region	.296	.227	1.305
Contact x Prop. Māori	.208	.071	2.945***
***Step 2***			
Intercept	5.498	.042	130.095***
Contact with Europeans	.031	.011	2.726*
Proportion of Māori in Region	.264	.222	1.190
Contact x Prop. Māori	.191	.067	2.864**
Gender	−.096	.084	−1.134
Income (log)	−.141	.058	−2.408*
Active Harm	−.024	.040	−.608
Cognitions of Rejection	−.048	.026	−1.857
Realistic Threat Perceptions	−.118	.026	−4.476***

**Note.** *p<.05, **p<.01, ***p<.001. All within-region effects were modeled as random. All variables were grand-mean centered.

**Table 3:** Model Statistics for New Zealander Europeans, Predicting Warmth Towards Māori.

	*b*	*se*	*t*
***Step 1***			
Intercept	4.470	.023	202.911***
Contact with Māori	.114	.018	6.421***
Proportion of Europeans in Region	.193	.216	.896
Contact x Prop. Europeans	.135	.133	1.020
***Step 2***			
Intercept	4.739	.022	216.203***
Contact with Māori	.117	.018	6.624***
Proportion of Europeans in Region	.071	.194	.367
Contact x Prop. Europeans	.043	.116	.372
Gender	−.128	.044	−2.943**
Income (log)	−.057	.033	−1.739
Active Harm	−.108	.025	−4.393***
Cognitions of Rejection	−.072	.016	−4.638***
Realistic Threat Perceptions	−.210	.015	−13.972***

**Note.** *p<.05, **p<.01, ***p<.001. All within-region effects were modeled as random. All variables were grand-mean centered.

### Analysis of New Zealander Europeans

For New Zealander Europeans, more hours with Māori friends predicted increased warmth toward Māori (*b* = .114, *se* = .018, *t* = 6.424, *p*<.001). The proportion of European participants' neighborhoods made up of fellow Europeans was not associated with warmth toward Māori (*b* = .071, *se* = .194, *t* = .367, *p* = .714) and did not moderate the effect of contact (*b* = .043, *se* = .116, *t* = .372, *p* = .710). The residual variance in warmth toward Maori was non-significant (*σ^2^* = .040, *se* = .071, *p* = .573).

### Analysis of Māori

For Māori, more hours with European New Zealander friends predicted increased warmth toward European New Zealanders (*b* = .031, *se* = .011, *t* = 2.726, *p* = .006). The proportion of Māori participants' neighborhoods made up of fellow Māori was not associated with warmth toward Europeans (*b* = .264, *se* = .222, *t* = 1.190, *p* = .234). As hypothesized, however, the main effect of outgroup contact was moderated by the proportion of ingroup members in the immediate neighborhood (*b* = .191, *se*  = .067, *t* = 2.864, *p* = .004). The residual variance in warmth toward Europeans was also non-significant (*σ^2^* = .036, *se* = .022, *p* = .104). We estimated conditional slopes for the moderated effect at 20% unit increases in the ethnic ingroup (Māori) proportion of the neighborhood in which participants resided.

As shown in Figure 1, spending time with New Zealander European (outgroup) friends had a stronger effect on warmth toward New Zealander Europeans for Maori living in regions with a higher proportion of other Māori (i.e., a lower proportion of *outgroup* members). The simple slope for the relationship between hours with New Zealander European friends and outgroup warmth was non-significant for Māori who lived in areas with only 1% of other Māori (*b* = −.003, *se* = .019, *t* = −.157, *p = *.875). However, this association was significant for Māori who lived in areas with 20% of other Māori (*b* = .033, *se* = .011, *t* = 3.011, *p* = .003), and stronger still for Māori who lived in areas with 40% of other Māori, 60% of other Māori, and 80% of other Māori (*b* = .072, *se* = .015, *t* = 4.747, *p*<.001; *b* = .110, *se* = .026, *t* = 4.186, *p*<.001; and *b* = .148, *se* = .039, *t* = 3.816, *p*<.001, respectively).

## Discussion

Most areas of the world (and in particular the Western world) are increasingly multicultural [18], [30]. As such, it is important to develop a holistic view of intergroup contact, looking at minority groups' perspectives as well as those of traditionally advantaged majority groups. In the present study, we examined the link between intergroup contact and outgroup attitudes in both majority and minority group samples. We proposed and found evidence of a wallpaper effect for minority group members: To the extent that minority group members were surrounded by a relatively high number of outgroup members in their neighborhood, contact appeared to lose its power to transform their intergroup attitudes. When minority group members' experiences reflected that of the majority group, however, and they lived surrounded by fellow minority group members, cross-group friendship displayed the classic pattern of prediction.

### Summary of findings

Our results reflected those found in previous studies [2]. While Māori who reported spending more hours with European friends also felt warmer towards New Zealander Europeans in general, the relationship was not as strong as the relationship between hours spent with Māori friends and warmth towards Māori for New Zealander Europeans. Critically, we could go some way towards explaining this asymmetry. As hypothesized, we found that neighborhood composition moderated the relationship between contact and outgroup warmth for Māori participants. Māori who lived in neighborhoods with a relatively low ratio of outgroup members (i.e., high ratio of fellow minority group members) showed the classic relationship between cross-group friendship and outgroup warmth – the more hours they spent with New Zealander European friends the more warm they felt towards New Zealander Europeans in general. Those who lived in neighborhoods with a very high ratio of outgroup members failed to show this association. This interaction emerged only for Māori (minority group members), and not for New Zealander Europeans (majority group members). Majority group members, as we have argued earlier in the paper, are almost *always* in the majority in terms of cultural and societal dominance, irrespective of the ethnic or racial composition of their neighborhood. Unlike minority group members, it would be very rare that the wallpaper of majority group members' existence would be patterned with outgroup faces, and so contact retains its potential to be remarkable and transformative. This is, we suggest, at the heart of the asymmetry. Were majority group members put in situations, however, in which this was reversed (i.e., they became minority group members), we would expect to see them display the same pattern of results found in our minority group sample.

Finally, for Māori the interaction predicted warmth towards European New Zealanders irrespective of how much active harm they reported experiencing, how concerned they were about being rejected on the basis of their race, or how much threat they perceived from majority group members. This lends weight to our proposition that outgroup proportion helps to explain the difference in majority and minority experiences of contact, over and above differing experiences of racism and perceptions of threat.

As stated previously, we did not expect, or find, a moderated relationship for majority group members. However, our primary hypothesis that majority group members living in regionally diverse neighborhoods would show decreased warmth towards minority group members was not supported. For them, neighborhood composition did not predict outgroup warmth. Our finding may be attributed to a number of factors. First, New Zealand has a different social history to many other Western countries, insofar as many Māori are relatively integrated into the national identity [31]. Both English and Māori are official languages in New Zealand, and it is a nation that is officially bicultural. The bicultural nature is ingrained in the New Zealand psyche – past research has found that European New Zealanders have an equally strong implicit association between both Māori and European faces and images of nationhood [32]. This is different from America and Australia, where White faces are most strongly implicitly linked to images of nationhood when compared to minority group faces [32].

It is possible then that the different social context in New Zealand explains the fact that regional diversity was not associated with lack of warmth. It should be noted, however, that neither was regional diversity linked to increased warmth. A hardline contact perspective would expect to see regional diversity predicting increased intergroup warmth in the absence of salient intergroup competition or division. We did not find this either – suggesting that while New Zealand has a more positive intergroup situation than many other comparable Western nations, it is not yet in the position where diversity predicts harmony, as is the case in certain areas of Germany and Italy [18], [22], [23].

### Neighborhood composition and the minority-majority asymmetry in contact effects

Past research consistently shows that minority group members show a weaker association between intergroup contact and intergroup attitudes than do majority group members [2]. A variety of explanations for this phenomenon have been suggested in the literature, but these remain largely untested. Explanations include the different histories of contact experienced by majority and minority groups, and present social inequality [2], [9], [33]. While we agree that all these factors are important in shaping how contact is differentially pursued and experienced by majority and minority group members, we proposed that a more basic factor could help to account for this varying pattern. Specifically, we argued that the proportion of one's neighborhood made up of fellow minority group members would moderate the contact-prejudice relationship in minority groups.

By virtue of their numerical minority, minority group members are exposed to majority group members more than the reverse [21], [24]. Part of what ensures contact works is that meeting an outgroup member is a remarkable circumstance that challenges previously held beliefs about the outgroup, and thus can overcome long-held anxieties and fears [11], [34]. While contact over time will become normal, and indeed, this is in part the way that contact works to alter attitudes, the process requires that it is sufficiently impactful to create attitudinal change. Minority group members are forced to have more – and more extended – contact with majority group members. Not only are they more likely to work with and live alongside majority group members, they are also more likely to be exposed to outgroup members through the media, TV shows and movies [21], [24]. In most of the Western world, as in our two samples, the “wallpaper” is White. This makes contact with minority group members inherently more likely to be memorable and transformative for majority group members than contact with majority group members is for minority group members. Thus we argued that we should find that the relationship between intergroup contact and intergroup attitudes for minorities is stronger the lower the proportion of (majority) outgroup members in one's neighborhood. Where the proportion of outgroup members is relatively low, contact should be as predictive for minority group members as it typically is for majority group members.

This is exactly what we found. For Māori (a minority group members in a Western nation), friendships with European people most strongly predicted increased warmth towards Europeans when participants lived in minority dense areas (i.e., areas with a relatively low proportion of Europeans). We therefore argue that outgroup proportion (or *opportunity for contact*) may be an important variable to examine when looking at the relationship between contact and warmth in minority group members.

We were aware, however, that it was possible that a third factor might explain any moderated relationship that we found between contact and outgroup proportion when predicting attitudes towards the outgroup. Specifically, past explanations of the asymmetry between minority and majority groups in responding to intergroup contact have relied heavily on the degree of racism and discrimination experienced by minority groups at the hands of majority groups [2], [8], [9]. In addition, past research has consistently shown that the presence of outgroup neighbors is linked to threat [15]–[17]. Doubtless, as we have argued in previous papers [11], [26], [34], these experiences shape and influence intergroup relations in multiple ways. In the current example, however, our effects held even when controlling for such factors as experiences of harm, cognitions of race-based rejection and perceived threat. If increased levels of racism or threat explained the weaker association for those living in “White” neighborhoods we should have seen our interaction disappear with the introduction of control variables. Instead, our effects held in all cases. Further to this, in our particular sample an examination of the association between variables reveals that for the minority group sampled in the present study (Māori), the presence of outgroup neighbors was actually associated with lower experiences of harm, fewer cognitions of race-based rejection, and decreased perceptions of threat. As such it is particularly unlikely that any one of these factors explains our effect.

This is not to say that the wallpaper effect necessarily fully explains the asymmetry observed between minority and majority groups. In fact, we would suggest that it is unlikely that this is the case. Multiple studies examining contact effects in minority groups show differing associations [35]–[37]. While all samples presumably varied in the extent to which they lived and worked surrounded by majority or minority group members, we do not see homogeneity in effects across samples. Rather, sometimes contact effects are non-existent or negligible, while other times they are large. It is likely that differing histories, current racial climates, and other factors might impact on whether contact works or not, or even further qualify the wallpaper effect. Thus, future research is needed to disentangle the process through which contact works in racially diverse and homogenous neighborhoods.

### Strengths, limitations and future directions

The present study employed large representative samples of majority and minority group members – this allowed us to test identical contact models in both to best compare the differential relationships between intra- and intergroup contact, minority group proportion, and warmth towards the outgroup. Our results help to explain the body of research that has found that minority group members typically show weaker associations between intergroup contact and warmth towards the outgroup than do majority group members.

Our data is cross-sectional, and while we feel that our causal model makes most sense theoretically, and further, that assumptions made in our introduction are borne out by the data, causality cannot be established. It would be possible, for example, to examine how neighborhood composition predicts warmth at different levels of cross-group friendship. Likewise, outgroup warmth may be having a reciprocal effect on hours spent with cross-group friends, or even where participants choose to live. Future research should attempt to clarify the causal nature of the observed relationship by longitudinally examining the association between cross-group friendship, racial and ethnic neighborhood composition and intergroup attitudes in both majority and minority groups. Likewise, our results should not be taken to indicate that in order to achieve maximum positive benefits of intergroup contact in minority groups minority dense neighborhoods are preferable. In fact, neighborhoods predominantly consisting of minority group members are typically economically disadvantaged [38]. Instead, our results help to explain how contact works for minority group members, and speaks to the necessity of targeting alternate ways in which negative attitudes about majority groups held by minority groups might be altered.

A final overarching point to be made is that aiming to improve minority group members' attitudes towards majority groups may not be the best way to increase social equality and intergroup reconciliation. While we know that majority group members' positive attitudes can translate into increased support for minority groups [11], the impact of increasing positivity towards majority groups in minority groups is ambiguous. Collective action models of social change suggest that for minority groups, a degree of anger towards the majority group is necessary to spur group-based action aimed at promoting the interests of the ingroup [39], [40]. In line with this, recent research shows that positive intergroup contact experienced by minority group members, and the resultant positivity directed towards the majority group, can prompt members of minority groups to overlook or underestimate real and pervasive discrimination [41], [42]. Likewise, minority group members who perceive the majority group to be rejecting actually endorse ingroup-favoring political action more than those who perceive them to be accepting [26].

### Conclusions

The contact literature is rich, varied, and one of the best articulated in social psychology. From it we can gather a multitude of information about the way in which contact works to reduce prejudice in majority group members (and the way that prejudice discourages majority group members from seeking out contact) [1]. Less understood, however, is the way in which contact works in minority groups, and in particular, why the relationship between contact and prejudice is consistently weaker in minority group samples [2]. In the present study, we go some way to explaining this phenomenon by demonstrating that minority group proportion qualifies the contact-outgroup warmth relationship for minority group members. Our results should also serve as a caution to social psychological theorists and those working in applied anti-prejudice. Models of prejudice reduction tested and supported among majority members cannot necessarily be generalized to minority members.
